# Epidemiology, Molecular Characterization and Antibiotic Resistance of *Neisseria meningitidis* from Patients ≤15 Years in Manhiça, Rural Mozambique

**DOI:** 10.1371/journal.pone.0019717

**Published:** 2011-06-10

**Authors:** Ana Belén Ibarz-Pavón, Luis Morais, Betuel Sigaúque, Inacio Mandomando, Quique Bassat, Ariel Nhacolo, Llorenç Quintó, Montse Soriano-Gabarró, Pedro L. Alonso, Anna Roca

**Affiliations:** 1 Centre de Recerca en Salut Internacional de Barcelona (CRESIB), Universitat de Barcelona, Barcelona, Spain; 2 Centro de Investigação em Saúde de Manhiça (CISM), Manhiça, Mozambique; 3 CIBER Epidemiología y Salud Pública (CIBERESP), Instituto de Salud Carlos III, Madrid, Spain; 4 Instituto Nacional de Saúde, Ministerio de Saúde, Maputo, Mozambique; 5 GlaxoSmithKline Biologicals, Rixensart, Belgium; Swiss Tropical and Public Health Institute, Switzerland

## Abstract

**Background:**

The epidemiology of meningococcal disease in Mozambique and other African countries located outside the “meningitis belt” remains widely unknown. With the event of upcoming vaccines microbiological and epidemiological information is urgently needed.

**Methods:**

Prospective surveillance for invasive bacterial infections was conducted at the Manhiça District hospital (rural Mozambique) among hospitalized children below 15 years of age. Available *Neisseria meningitidis* isolates were serogrouped and characterized by Multilocus Sequence Typing (MLST). Antibiotic resistance was also determined.

**Results:**

Between 1998 and 2008, sixty-three cases of confirmed meningococcal disease (36 meningitis, 26 sepsis and 1 conjunctivitis) were identified among hospitalized children. The average incidence rate of meningococcal disease was 11.6/100,000 (8/100,000 for meningitis and 3.7/100,000 for meningococcemia, respectively). There was a significant rise on the number of meningococcal disease cases in 2005–2006 that was sustained till the end of the surveillance period. Serogroup was determined for 43 of the 63 meningococcal disease cases: 38 serogroup W-135, 3 serogroup A and 2 serogroup Y. ST-11 was the most predominant sequence type and strongly associated with serogroup W-135. Two of the three serogroup A isolates were ST-1, and both serogroup Y isolates were ST-175. *N. meningitidis* remained highly susceptible to all antibiotics used for treatment in the country, although the presence of isolates presenting intermediate resistance to penicillin advocates for continued surveillance.

**Conclusions:**

Our data show a high rate of meningococcal disease in Manhiça, Mozambique, mainly caused by serogroup W-135 ST-11 strains, and advocates for the implementation of a vaccination strategy covering serogroup W-135 meningococci in the country.

## Introduction

*Neisseria meningitidis* is a major cause of meningitis and septicaemia worldwide that often leaves survivors with severe sequelae [1]. Effective vaccination strategies have contributed to maintain the rate of meningococcal disease (MD) low in industrialized countries [2], [3]. However, resource-poor countries continue to struggle with this devastating disease. In the African meningitis belt, which comprises all countries stretching across Africa from Ethiopia to Senegal [4], incidence rates reach over 55/100,000 population [5] and can surpass 1,000/100,000 during the large-scale epidemics that are a unique characteristic of the belt [6]. Historically, disease outbreaks in Africa have been caused by serogroup A [7], but a Hajj-related serogroup W-135 strain emerged as an important cause of disease in 2000 [8], [9], [10], [11] and serogroup X meningocci have also been recently associated with several outbreaks [12], [13], [14].

Despite concerns of a possible expansion of the meningitis belt [15], data on MD from African countries outside this former area are still scarce. Climate conditions and meteorological disasters make Mozambique a country likely to suffer outbreaks of MD, and various environmental modeling systems have forecasted epidemics in northern and southern areas that appear yet to be materialized [16], [17], [18]. The overall burden of disease reported from Mozambique seems low, but the true nationwide incidence remains unknown, as cases are often unreported despite the existence of a national reporting system for communicable infectious diseases [19], [20]. In Manhiça, a rural village in Southern Mozambique, invasive bacterial infections are monitored by the *Centro de Investigaçâo em Saude da Manhiça* (CISM) among children hospitalized at the Manhiça District Hospital (MDH) since 1998 [21], [22], [23]. Surveillance data revealed *Streptococcus pneumoniae* and *Haemophilus influenzae* type b as the major cause of acute bacterial meningitis in Manhiça among children ≤15 years of age, with *N. meningitidis* being the third. However, whereas the prevalence pneumococcal and *Hib* meningitis remained stable, the incidence of meningococcal meningitis experienced a significant increase in recent years [21], [23]. Additionally, a study published by Zimba *et. al.* suggested that the meningococcus is the first cause of meningitis in the capital, Maputo [24].

This article presents data on the incidence, epidemiology, molecular characteristics and antibiotic susceptibility of meningococcal isolates obtained from patients admitted to the MDH over a period of 11 years.

## Materials and Methods

### Study site and population

The study was conducted in the Manhiça district, located 80 km north of Maputo and with an estimated population of 143,000 inhabitants, of which an estimated 26% are under 15 years of age [25]. The climate is sub-tropical with a warm and rainy season between November and April, and a cool and dry season during the rest of the year. Malaria is endemic throughout the year, peaking between December and March. The point-prevalence of HIV among pregnant women in 2004 was estimated as 21%, but an increase from 18.4% in 2003 to 29% in April 2005 was observed [23], [26].

A continuous Demographic Surveillance System (DSS) runs at the CISM since 1996. Initially, the DSS covered a 100 Km^2^ area and included 35,000 inhabitants. In August 2002 the area was expanded to 400 Km^2^ and the population within the DSS increased to 70,000. Currently, the area covers 500 Km^2^ and includes 82,000 individuals. Information on births, deaths and migration movements were collected and updated quarterly until 2000, and twice a year since. A unique permanent identification number issued to all subjects living within the DSS area and recorded in all hospital attendances at the MDH allows to link morbidity and demographic data.

### Case identification

Since 1997, a standardized clinical questionnaire is filled in upon hospital admission for all patients, and outcome data recorded at discharge [21], [23]. All children with fever (axillary temperature ≥37.5) or with a history of fever are tested for malaria parasites in blood. Blood cultures were performed routinely since 1998 to all admitted children <2 years of age, for older children with axillary temperature >39°C, and for those presenting symptoms compatible with sepsis or neurological impairment. Prior to 2006, lumbar punctures (LP) were performed to all children presenting neurological signs compatible with meningitis at the clinician's criterion, and cerebro-spinal fluid (CSF) samples were processed only for bacterial isolation. In January 2006, and enhanced meningitis surveillance was established and criteria for the collection of CSF on cases of suspected meningitis were standardized [21]. Additionally, LP was since performed to all neonates (<28 days of age) admitted to hospital with fever or suspected neonatal sepsis. CSF samples were processed following a standardized protocol as described in Roca *et. al.* [21].

### Ethics

The implementation of the enhanced meningitis surveillance was approved by the Mozambican National Bioethics Committee and the Institutional Review Board of the Hospital Clinic of Barcelona. The ethics committee did not require informed consent from patients as the surveillance involved improvements in clinical criteria and management of cases with suspected meningitis that were incorporated into the hospital's standard operational procedures.

### Case definition

Laboratory-confirmed meningococcal disease was defined as the presence of *N. meningitidis* in a normally sterile body fluid. Meningococcemia was defined as isolation of *N. meningitidis* from blood and not from CSF. Meningitis was defined as (i) isolation of the bacterium in CSF regardless of its presence in blood; or (ii) positive latex agglutination for *N. meningitidis* antigens in a purulent CSF. Purulent CSF was determined by a turbid appearance, a leukocyte count of ≥100×10^6^/L or a leukocyte count of ≥10–99×10^6^/L in combination with either a glucose level of <40 mg/dL or the presence of protein as determined by the Pandy test [27]. Meningococcal conjunctivitis was defined as the isolation of *N. meningitidis* from a purulent ophthalmic exhudate.

### Treatment guidelines

Mozambique national recommendations for the treatment of suspected meningitis include the administration of intravenous chloramphenicol (100 mg/Kg/day) or combinations with intravenous penicillin G (2 500.000 ui/Kg/day) plus gentamicin (daily 5–7.5 mg/kg). Infants under 2 months are treated with ampicillin (200 mg/Kg/day) and gentamicin (5 mg/Kg/day). Treatment is reassessed once bacteriological results and antibiotic susceptibility are known. When available, ceftriaxone (100 mg/Kg/day) is used in all confirmed meningitis cases until the antibiotic susceptibility pattern is obtained. Direct contacts of MD patients are administered rifampicin for prophylaxis (20 mg/kg/day for 2 days) [21].

### *N. meningitidis* identification

Gram-negative diplococci, oxidase, catalase positive and capable of fermenting glucose were identified as *N. meningitidis*. Isolates were stored at −80°C in a vial containing skim milk. Additional identification tests (*e.g.* latex agglutination) were performed for the purpose of this study upon culture when in doubt.

### *N. meningitidis* culture and DNA extraction

Isolates were retrieved from the freezer and cultured in Columbia agar base with horse blood. Plates were incubated at 37°C in an atmosphere of 5% CO_2_. Growth was checked after 12 h, and a single colony was re-plated following the same procedure. DNA extractions were performed using the Qiagen DNA Mini Kit (Qiagen).

### Serogroup determination

Since 2006, latex agglutination test for serogroups A, B, C and W-135/Y (Pastorex® test, BioRad) were routinely performed in all CSF samples rendering a positive gram stain or cell count. A previously published Polymerase Chain Reaction (PCR) assay was used to ascertain the serogroup in all viable isolates [28].

### MultiLocus Sequence Typing (MLST)

Viable isolates were characterized by MLST as described by Maiden *et al.* [29]. The internal fragments of seven housekeeping genes were amplified by PCR and the forward and reverse strands were sequenced at the Macrogen Inc. facility in Seoul, Korea. Allele designations were determined by querying the *Neisseria* MLST database (www.pubmlst.org/neisseria). New MLST profiles were sent to the database curator at the University of Oxford for designation.

### *porA* and *fetA* variable region typing

Following the guidelines of the European Monitoring Group for Meningococci (EMGM), the variable regions of the antigens *porA* and *fetA* were determined as previously described [30] and designated by querying the databases on www.neisseria.org/nm/typing.

### Antibiotic susceptibility testing

Antibiotic susceptibility and Minimum Inhibitory Concentrations (MICs) for chloramphenicol, penicillin G, gentamicin, rifampicin and trimethoprim-sulfamethoxazole (cotrimoxazole) were determined by Etest® (AB Biodisk, Solna, Sweden) following the manufacturer's recommendations, and results were interpreted according to NCCLS standards.

### Data Management and Analysis

Questionnaire data are routinely double-entered and stored in a proprietary database following the standard procedures at CISM. Incidence rates ratios (IRR) and 95% CI rates were calculated among children living within the DSS area at the time of the disease onset using the number of laboratory-confirmed cases of meningococcal disease, meningitis and meningococcemia as defined previously, and on positive blood cultures regardless of evidence of *N. meningitidis* in CSF. Time at risk was calculated as the number of person years at risk since the beginning of the time at risk until the end of follow-up, 15 years of age, migration or death, whatever occurs first. All analyses were performed using Stata/SE 11 for Windows.

## Results

A total of 42,374 children under15 years of age were admitted to the Manhiça district hospital between 1^st^ of January 1998 and 31^st^ of December 2008 (mean annual admission 3,856), of which 2,587 (6.1%) were hospitalized with suspected meningitis. Sixty-three cases (2.4%) of MD were identified among these patients. The median age of the cases was 30 months and 54% were male. By age groups, 30.2% of the cases occurred among children <1 year, 34.9% were aged 1–<4 year, 23.8% 4–<10 years and those aged 10–15 years represented 11.1% of patients.

**Figure 1:**
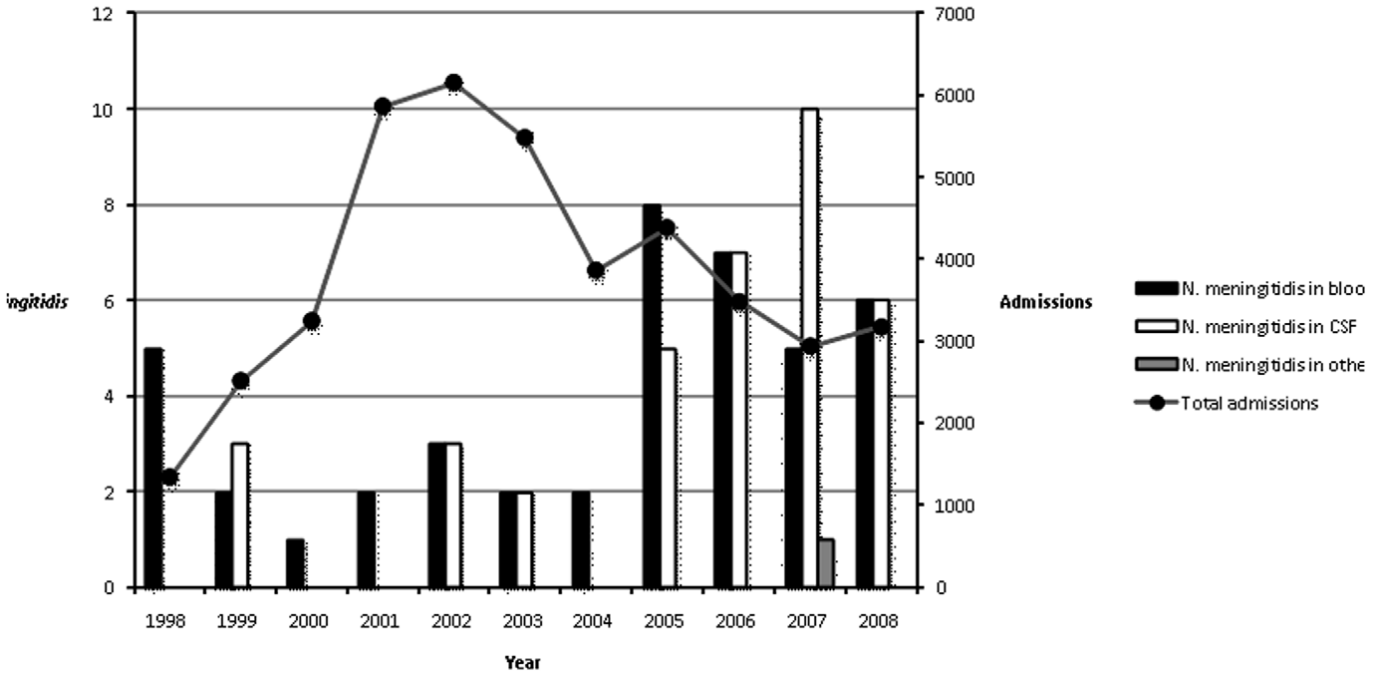
Total number of *N. meningitidis* isolated from blood, CSF and other sterile fluids *vs.* total number of admissions to the MDH for the period 1998–2008.

Thirty-six cases presented meningococcal meningitis (57%), six of which were negative by culture but could be detected by latex agglutination test. Seventeen (27%) of these patients had concurrent meningococcemia. Twenty-six patients (41%) presented meningococcemia alone. One isolate was obtained from the ophthalmic exudate of a purulent conjunctivitis case (Figure 1).

### Incidence rates

Minimum community-based incidence rates for meningococcal disease among children ≤15 years of age living within the study area were calculated (n = 35). Table 1a shows the overall incidence rates for meningococcal disease and those based on the number of positive blood cultures, as well as the rates for meningitis and meningococcemia in different age groups. The overall incidence rate of meningococcal disease for the period 1^st^ January-1998 – 31^st^ December 2008 was 11.6/100.000 child-year at risk. The incidence of meningococcal meningitis doubled that of meningococcemia (8/100.000 *vs.* 3.7/100.000). The highest incidence rates on all presentations of meningococcal disease, were seen among children <4 years of age, with rates halving among those aged 4–<10 years, and rising again among teenagers.

**Table 1:** Minimum Incidence rates of meningococcal disease according to age groups, disease presentation and year of surveillance. Only cases from children residents from the DSS are included.

	Overall rate			<1 years			1–<5 years			5–<15 years		
	Number of patients		Rates (95% CI)	Number of patients		Rates (95% CI)	Number of patients		Rates (95% CI)	Number of patients		Rates (95% CI)
Meningococcal disease	35		11.6 (8.4–16.2)	7		26.9 (12.8–56.4)	14		15.1 (9–25.5)	14		7.7 (4.6–13)
Meningitis	24		8 (5.4–11.9)	2		7.7 (1.9–30.7)	11		11.9 (3.3–10.9)	11		6 (3.3–10.9)
Bacteraemia	11		3.7 (2–6.6)	5		19.2 (8–46.1)	3		3.2 (1–10)	3		1.6 (0.5–5.1)
Positive blood culture	25		8.3 (5.6–12.3)	6		23 (10.4–51.3)	10		10.8 (5.8–20.1)	9		4.9 (2.6–9.5)

	Overall			Meningitis			Bacteraemia			Blood culture		
Time period	Number of patients		Rates (95% CI)	Number of patients		Rates (95% CI)	Number of patients		Rates (95% CI)	Number of patients		Rates (95% CI)
1998–2001	4		6.5 (2.4–17.3)	1		1.6 (0.2–11.5)_	3		4.9 (1.6–15.1)	4		6.5 (2.4–17.3)
2002–2004	6		6.9 (3.1–15.3)	4		4.6 (1.7–12.2)	2		2.3 (0.6–9.2)	5		5.7 (2.4–13.8)
2005–2008	25		16.5 (11.1–24.4)	19		12.5 (8–19.6)	6		4 (1.8–8.8)	16		10.5 (6.5–17.2)

Disease rates fluctuated over the years (table 1b), but a significant increase on the incidence of MD occurred in 2005 and was sustained until the end of the study period.

### Clinical data on meningococcal disease

Among the 36 meningitis cases, the average time from disease onset to hospitalization, measured as the number of days from the referred onset of fever, was 3 days, and the mean hospitalization time was 8 days. A total of 27 (75%) meningitis patients had fever at the time of hospitalization (mean temperature 38°C). At least one episode of convulsions occurred in 10 (28%) of these patients. Neck stiffness was present in 16 out of 28 (57%), and a bulging fontanellae was observed in 3 children out of the 8 for whom this criteria was applied (age <1,5 year) Anorexia and vomiting were present in 13 (36%) and 10 (28%) meningitis patients respectively. Two patients (8%), aged 2 and 10 years respectively, died of meningitis within 24 h of presenting fever, and one was transferred to the Central hospital in the capital, Maputo.

For the 26 meningococcemia cases, the average time from the start of fever to hospital attendance was 2 days and the mean hospitalization time was 5 days. A total of 21 (78%) of these patients presented fever at the time of hospitalization (mean temperature 38.2°C). None of the patients diagnosed with meningococcemia presented neck stiffness; however, three patients experienced fits (12%). Vomiting and anorexia was seen in 19 (73%) and 5 (19%) of these patients respectively. One 5 years old child (4%) with meningococcemia died four days after disease onset and another one absconded hospital and final outcome is not available (Table 2).

**Table 2:** Clinical symptoms associated with meningococcal meningitis and bacteraemia among patients treated at the MDH between 1998–2008.

	Meningococcemia cases (%) (n = 26)	Meningitis cases (%) (n = 36)
Disease onset	2 days	3 days
Hospitalization time	5 days	8 days
Fever at arrival	21(78)	27(75)
Mean temperature at arrival	38.2°C	38°C
Convulsions	3(12)	10 (28)
Neck stiffness	0	16 (44)
Bulging fontanelle (children <1 year)	0	**3 (38)
Anorexia	5(19)	13(36)
Vomiting	19(73)	10(28)
Malaria parasites*	9(39)*	5 (16)
Death	1(4)	2(8)
Unknown outcome	1(4)	1(3)

*n = 23 for meningococcemia and n = 31 for meningitis).

**out of 8 children <1 year of age.

Data on malaria parasitemia was available for 31(86%) meningitis and 23 (88%) of meningococcemia cases. A total of 5 meningitis (16% of 31 cases with data available) and 9 meningococcemia patients (39% of 23 with data available) showed evidence of *Plasmodium falciparum* parasites in their blood.

The conjunctivitis case was 19 days old newborn presenting with dehydration and fever for one day before hospitalization. The patient had a negative blood culture and was also negative for malaria.

### Isolate collection

A total of 63 cases of laboratory-confirmed MD were admitted at the MDH over the surveillance period. Among those, 48 isolates (26 from blood, 21 from CSF and one from a purulent ophthalmic exudate) obtained from 37 patients were available for genetic characterization and antibiotic susceptibility testing. Isolates obtained from blood and CSF from the same patient were available for 11 cases.

### Meningococcal serogroups

Serogroup by latex was determined for 21 of the 36 cases (55%) of laboratory-confirmed meningitis. Serogroup was determined by PCR [31] for all 37 cases of meningococcal disease from whom isolates were available. In all cases for which two isolates were available, serogroup results on blood and CSF were concordant. Latex agglutination and PCR results were concordant in all but one sample, which was reported non groupable on agglutination, but positive for W-135 by PCR. Latex agglutination results were available for 6 additional cases from whom the original isolate was no longer available for PCR confirmation.

For the total 43 cases of meningococcal disease for which the serogroup of the causative strain was determined (21 by latex and PCR, 16 by PCR and 6 by latex alone), 38 (88%) were caused by serogroupW-135, 3 (7%) were serogroup A, and the remaining 2 (5%), serogroup Y. Serogroup W-135 isolates were first detected in 1998 and continued to be seen throughout the years with a substantial upsurge since 2005. Serogroup A isolates were seen in 1999, 2003 and 2006. Serogroup Y isolates were detected in 1999 and 2007 (Figure 1).

### Genetic characterization

From the 48 *N. meningitidis* isolates characterized by MLST *porA* and *fetA*, 26 (54%) were isolated from blood samples, 21 (44%) were obtained from CSF, and 1 (2%) came from an eye exudate. Isolates came from a total of 37 cases of disease.

Five different STs belonging to 3 different clonal complexes were identified among the isolates. ST-11 complex was the cause of disease in 31 patients (84% of cases for which MLST was performed). Two novel STs were detected and assigned as ST-7464 and ST-7465. ST-7464 differs in two loci from ST-11 and belongs to this clonal complex. The ST-7465 isolate obtained from the conjunctivitis patient does not belong to any of the known clonal complexes to date, but its genetic profile shares 6 of the 7 loci with *Neisseria gonorrhoeae* isolates listed on the pubmlst database. The seventh allele, *fumC*3, is in common with ST-11 *N. meningitidis*. This isolate, along with all ST-11 complex isolates belonged to serogroup W135. ST-1 and ST-175 were each identified in two patients and had serogroup A and serogroup Y capsule respectively.

A total of six different *porA* types were found among the isolates, all but one belonging to VR1 family 5 and VR2 family 2. The ST-7465 isolate obtained from the conjunctivitis sample had a *porA* type unrelated to those found among the other isolates and found to be common to *N. gonorrhoeae*.

Four different *fetA* variants were identified among the strains, three of which belonged to family five, and one to family one, with the latter being exclusively associated to ST-11. It was not possible to determine the *porA* and *fetA* variants for the ST-7464 isolate (Table 3).

**Table 3:** Strains of *N. meningitidis* isolated by year from patients of meningococcal disease attended at the MDH from 1998–2008.

	Year											
Strain type	1998	1999	2000	2001	2002	2003	2004	2005	2006	2007	2008	Total
**ST-1 complex**												
A:P1.5,2-10:F5-1:ST1		1				1						2
**ST-11 complex**												
W135:P1.5,2:F1-1:ST11	1						1	9	10	7	11	39
W135:P1.5,2-2:F1-1:ST11				1						1		2
W135:P1.5,2-38:F1-1:ST11											1	1
W-135: P1.ND,ND: F-ND: ST-7464										1		1
**ST-175 complex**												
Y:P1.5-1, 2-2: F5-8: ST-175		1								1		2
**Unassigned**												
W-135:P1.18-10,43:F5-24: ST-7465										1		1

### Antibiotic susceptibility

One patient diagnosed with meningococcemia in 1998 harboured a strain that was resistant to chloramphenicol, rifampicin, penicillin and trimethoprim-sulfamethoxazole. All other isolates were susceptible to chloramphenicol, rifampicin and gentamicin. One patient presented a penicillin resistant strain, and there was one case caused by a strain with intermediate resistance to penicillin. Twenty-on patients had isolates with intermediate resistance to trimethoprim-sulfamethoxazole and 2 patients harboured strains that were fully resistant to this drug. When blood and CSF isolates from the same patient were available, results were concordant for all antibiotics in all but one of the 11 patients, who appeared to have an isolate resistant to trimethoprim-sulfamethoxazole in CSF but susceptible in blood (Table 4).

**Table 4:** Susceptibility testing results for 37 invasive meningococcal disease patients.

	R≥	I	S≤
Chloramphenicol	1*		36
Rifampicin	1*		36
Penicillin	2*	1	34
Trimethoprim-Sulfamethoxazole	2*	21	14
Gentamicin	37		

*One isolate was resistant to all these drugs.

## Discussion

These data represent the first published study specifically on the characterization and incidence of meningococcal disease in Mozambique. The minimum incidence rates reported here are unusually high for an area situated outside the meningitis belt but yet below the high rates reported from the belt [5], [32]. However, our data underestimate the real community burden, as we presume that many additional cases never reached the hospital. Prior to 2006, LPs were rarely performed to children with no obvious signs of meningitis [27]. The standardisation of clinical criteria to identify suspected cases of meningitis in 2006 will have resulted in more cases being identified and therefore incidence rates calculated from 2006 onwards are expected to be more accurate. However, blood culture procedures have remained unchanged over the study period and incidence rates estimated from the number of positive bloods are comparable from year to year, which allows to asses an increase in the incidence of MD since 2005.

The highest incidence of meningococcal disease in the Manhiça district was seen among infants <1 year. Incidence rates among infants in Manhiça were seen to be similar to those reported from South Africa [33]. Serogroup W-135 meningococci have been associated with high rates of disease among the youngest, whereas serogroup A appears to affect more frequently older children and adults [10], [33]. The high prevalence of serogroup W-135 over serogroup A in our population might explain why the median age of MD cases in Manhiça is low.

The meningococcal strain responsible for most cases was the same that caused the Hajj-related global outbreak of MD in 2000 [8] and to which various epidemics in the meningitis belt were attributed to [10], [34], [35]. This strain was also associated to a rise in endemic disease and a high mortality in neighbouring South Africa [33]. The upsurge of this strain among MD patients during 2005 and 2006 was similar to that described in South Africa [33], [36], and might be explained by continuous migration movements young males from Manhiça to the neighbouring country in search of work.

The overall mortality rate for the 11-year period due to MD in Manhiça was low (5%, 3 out the 61 cases with known outcome) compared to the average 8% mortality seen among other African countries [37]. This also contrasts with the high case-fatality rates of 22% reported in South Africa, associated with ST-11 W-135 meningococcemia [33]. It is likely that some of the more severe cases of MD never reached hospital as it has been reported that 54% of deaths of children ≤15 years in the Manhiça area occur outside health facilities [38].

Since the Hajj-related outbreak of 2000, disease caused by W-135 meningococci has became endemic in many sub-Saharan African countries and caused sporadic epidemics. Furthermore, meningococcal carriage studies in sub-Saharan Africa have detected a high prevalence of the W-135:P1.5,2:ST-11 meningococcal strain among asymptomatic carriers [39], [40] which has led to debates on the necessity of including W-135 as part of any vaccination strategy being developed for this region of the world. Our data fully support the need for such vaccine, specially among African countries outside the meningitis belt, where the epidemiology of the disease and disease-causing strains differs greatly from that observed in the meningitis belt.

Serogroup A was the cause of disease in 3 (7%) of the cases, and the two viable isolates were characterized as A:P1.5,2-10:F5-1:ST1. Disease caused by this serogroup is associated with older infants and adolescents [10], [33], and although it is likely that our study missed some cases occurred among those over the age of 15, it is unlikely that data would significantly change our conclusions regarding the prevalence of this serogroup in the south of the country. However, data in the north of Mozambique should be needed to ascertain the burden of serogroup A.

The fact that the two cases of meningococcal disease caused by Y:P1.5-1, 2-2: F5-8: ST-175 are separated in time and location could suggest that meningococcal disease caused by this strain would be an sporadic event rather than a circulating endemic strain. However, the identification of one case caused by the newly detected W-135:ST-7564, which is identical to ST-11 except for the two alleles that shares with ST-175, *abcZ* and *aroE*, evidences recombination between ST-11 and ST-175 meningococci and hence long-term contact between these two STs. Asymptomatic carriage of ST-175 was seen in the Gambia [39], Niger [41]and Burkina-Faso [40] and is not unreasonable to suspect that this strain also circulates among asymptomatic carriers in Manhiça.

Neonatal conjunctivitis is a rare and under-investigated presentation of disease caused by *Neisseria* pathogenic species. The discovery of the ST-7465 strain, a w-135 capsulated meningococcus presenting a genetic profile that suggests it might be the result of recombination between *N. meningitidis* and *N. gonorrhoeae* proves that this phenomenon can occur, but is rare as they do not often converge in the same environment. It is likely the newborn acquired the recombinant strain on the birth canal [42].

*N. meningitidis* isolated from cases in the Manhiça district remained generally susceptible to those antibiotics commonly administered for the treatment of meningitis in Mozambique. However, meningococci with resistance to penicillin were detected among our isolates. Such isolates were also detected in South Africa, but are rarely reported from anywhere else in the continent [43]. As our work reveals, this could be due to a lack of data and their presence should not be ruled out in other African countries. A total 52.1% of the isolates tested presented intermediate or full resistance to cotrimoxazole. The widely-extended use of sulphonamides (Sulphadoxine-pyrimethamine) in Mozambique as treatment for uncomplicated malaria during most of the study period or as intermittent preventive treatment among pregnant women, and the use of cotrimoxazole as prophylaxis against *Pneumocystis jirovecii* pneumonia in HIV positive patients have resulted in the selection of resistant bacterial strains. This phenomenon has also been observed among pneumococci and non-typeable *Salmonella* isolates in the area [27].

Data generated from this long surveillance provide very strong evidence for the need to evaluate the introduction of W-135 meningococcal vaccines in countries outside the meningitis belt such as Mozambique. However, data on the epidemiology of MD from Northern Mozambique, where population and migration movements are different from those in the South, and data on disease on a wider age-range are necessary to support our findings.
